# Identification and verification of the pyroptosis-related prognostic signature and its associated regulatory axis in bladder cancer

**DOI:** 10.3389/fcell.2022.912008

**Published:** 2022-08-31

**Authors:** Yaofen Tu, Xiaodi Ding, Zujie Mao

**Affiliations:** ^1^ Department of Urology, Zhejiang Provincial People’s Hospital, Affiliated People’s Hospital, Hangzhou Medical College, Hangzhou, China; ^2^ Department of Rehabilitation, Zhejiang Provincial People’s Hospital, Affiliated People’s Hospital, Hangzhou Medical College, Hangzhou, China

**Keywords:** pyroptosis, bladder cancer, tumor microenvironment, prognostic signature, SNHG14

## Abstract

**Background:** Pyroptosis is an inflammatory form of cell death triggered by certain inflammasomes. Accumulating studies have shown the involvement of pyroptosis in the proliferation, invasion, and metastasis and prognosis of cancer. The prognostic value of pyroptosis-related genes (PRGs) and their association with immune infiltration in bladder cancer have not yet been elucidated.

**Methods:** We performed a comprehensive analysis of the prognostic value and immune infiltrates of PRGs in bladder cancer using the TCGA dataset. qRT-PCR was also performed to verify our result.

**Results:** Among 33 PRGs, 14 PRGs were upregulated or downregulated in bladder cancer tissue versus normal tissue. We also summarized copy number variations and somatic mutations of PRGs in bladder cancer. By using consensus clustering analysis of PRGs with prognostic significance, we divided the bladder cancer cohort into two subtypes significantly by different prognosis and immune infiltration. Using the LASSO Cox regression analysis, a prognostic signature including six PRGs was constructed for bladder cancer and the patients could be classified into a low- or high-risk group. Interestingly, this prognostic signature had a favorable performance for predicting the prognosis of bladder cancer patients. Moreover, further analysis demonstrated a significant difference in gender, tumor grade, clinical stage, TNM stage, immunoScore, and immune cell infiltration between the high- and low-risk groups in bladder cancer. We also identified an lncRNA SNHG14/miR-20a-5p/CASP8 regulatory axis in bladder cancer by constructing a ceRNA network.

**Conclusion:** We identified a PRG-associated prognostic signature associated with the prognosis and immune infiltrates for bladder cancer and targeting pyroptosis may be an alternative approach for therapy. Further *vivo* and *vitro* experiments are necessary to verify these results.

## Introduction

Bladder cancer is the ninth-most common cancer globally, resulting in over 210,000 deaths every year (Antoni et al., 2017; Sung et al., 2021). In China, approximately 42,973 patients died of this disease in 2022 (Xia et al., 2022a). As one of most deadly diseases, bladder cancer is characterized by a high rate of recurrence, committing patients to long-term invasive surveillance (Lenis et al., 2020; Lu et al., 2021). Despite some factors, including advanced age, male sex, and cigarette smoking, have been identified to be involved in the oncogenesis of bladder cancer, the specific mechanisms of bladder cancer have not yet been fully elucidated (Lenis et al., 2020). Little progress had been made in the past 30 years in the treatment for bladder cancer and only a limited range of therapeutics could be provided for bladder cancer patients (Grayson, 2017). The prognosis of bladder cancer patients varies greatly, with the 5-year overall survival (OS) rate ranging from 5% (patients in the advanced stage) to 90% (patients in the early stage) (Patel et al., 2020). With the development of molecular pathology, targeted therapies and immunotherapies, prognostic gene signatures based on the reclassification of bladder cancer subgroups were expected to be the most promising prognostic biomarkers for bladder cancer (Alifrangis et al., 2019). However, these studies were still at the molecular level and had not yet been applied in clinics.

Pyroptosis is an inflammatory form of cell death triggered by certain inflammasomes, leading to the cleavage of gasdermin D (GSDMD) and activation of inactive cytokines such as IL-18 and IL-1β (Fang et al., 2020). Accumulating studies have highlighted the significant role of pyroptosis in cardiovascular diseases and diabetic nephropathy (Zhaolin et al., 2019). Moreover, a pyroptosis-related gene signature could serve as a prognosis biomarker for different types of cancers, including ovarian cancer (Ye et al., 2021). The crosstalk between pyroptosis and immune infiltration had been also reported in tumor (Tang et al., 2020). However, the prognostic value of pyroptosis-related genes (PRGs) and their association with immune infiltration in bladder cancer have not yet been fully elucidated.

With the development of gene sequencing technology and the establishment and improvement of large tumor databases such as The Cancer Genome Atlas (TCGA), it is possible to systematically study the role of PRGs in tumors. In our study, we systematically and comprehensively investigated the potential role of PRGs in the prognosis and immune infiltration in bladder cancer. The result of the current study may provide additional data for prognostic biomarkers and therapy targets for bladder cancer.

## Materials and methods

### Datasets and preprocessing

The RNA sequencing (RNA-seq) dataset, somatic dataset, and clinical dataset of 414 bladder cancer patients was extracted from the TCGA database on 2 March 2021 and the clinicopathological parameters of the TCGA bladder cancer cohort patients are provided in Supplementary Table S1. Furthermore, the UCSC Xena website was used for the downloading copy number variation (CNV) data. R (version 4.0.5) project and R Bioconductor packages were utilized in dataset processing. After normalizing to transcripts per kilobase million (TPM) values, the RNA-seq data were used for further analysis.

### Identification of gene expression and genetic mutation of pyroptosis-related genes

Based on previous studies, we obtained 33 PRGs (Latz et al., 2013; Ye et al., 2021) (Supplementary Table S2). The “limma” and “reshape2” packages in R were utilized to detect the expression of PRGs in bladder cancer. Moreover, the “maftools” package in R was used to show the mutation frequency and oncoplot waterfall plot of 33 PRGs in bladder cancer. Moreover, the locations of 33 PRGs on chromosomes in CNV alteration were visualized by using the “RCircos” package.

### Gene Ontology and Kyoto Encyclopedia of Genes and Genomes pathway analyses

We then submitted these 33 genes into DAVID (https://david.ncifcrf.gov), a web-portal, for a comprehensive functional annotation analysis. The result was downloaded and visualized using the “ggplot2” package. It was noteworthy that biological process (BP), cellular component (CC), and molecular function (MF) were included in the Gene ontology (GO) analysis.

### Consensus cluster analysis

Cox regression analysis was performed to identify those PRGs with significant prognosis, and these PRGs were selected for further analysis. We then performed a consensus cluster analysis using the “ConsensusClusterPlus” package (1,000 iterations and a resample rate of 80%). Moreover, the gene-expression patterns between each subtype of the TCGA bladder cancer cohort were shown with the “pheatmap” package.

We then explored the difference of each subtype of TCGA bladder cancer cohort in the immune infiltration using the ESTIMATE algorithm, including immunoScore, StromaScore, ESTIMATEScore, and the abundance of 22 immune cell types.

The IPS score was downloaded from TCIA (https://tcia.at) and the IPS scores in the two clusters were visualized with the “ggpubr” package.

### Construction of the prognostic model based on pyroptosis-related genes

Based on those PRGs with prognostic significance identified with the Cox regression analysis aforementioned, we then constructed a prognostic model with the LASSO Cox regression analysis. Ultimately, a total of six genes and their coefficients were retained and the risk score was calculated after centralization and standardization as follows: Risk Score = GSDMB expression × (−0.0300) + CASP9 expression × (−0.1129) + GSDMD expression × (−0.0042) + CASP8 expression × (−0.0243) + AIM2 expression × (−0.0023) + CASP1 expression × (−0.0090). Bladder cancer patients in the TCGA cohort were separated into two subgroups (low- and high-risk) based on the risk score. A Kaplan–Meier analysis was conducted to evaluate the difference between these two subgroups in OS time. Moreover, an ROC analysis was conducted to evaluate the predictive accuracy. Univariate and multivariable Cox regression analyses were conducted to identified the factors affecting the prognosis of bladder cancer patients. Moreover, we also evaluated the correlation between the risk score and clinical characters and the abundance of 22 immune cell types by employing the “ggplot2” package.

### Construction of the CeRNA network

Search Tool for the Retrieval of Interacting Genes (STRING) was employed to construct a PPI network using the aforementioned six PRGs to identify the hub genes. This was followed by the identification of the miRNA target of the hub gene using StarBase (http://starbase.sysu.edu.cn/) and TarBase (https://carolina.imis.athena-innovation.gr). We also employed StarBase (http://starbase.sysu.edu.cn/) and LncBase (https://carolina.imis.athena-innovation.gr) to explore the lncRNA targets interacting with the miRNA target identified previously. Moreover, the expression and prognostic values of these miRNAs and lncRNAs were detected with the TCGA bladder cancer dataset.

### qRT-PCR

Approved by the Ethics Committee of Zhejiang Provincial People’s Hospital, a total of 50 bladder cancer tissues and normal bladder tissues were extracted from the patients. Each patient signed informed consents. Histological diagnosis was performed by three experienced pathologists according to the 2010 American Joint Committee on Cancer (AJCC) staging system. TRIzol reagent (Invitrogen) was utilized in the isolation of total RNA of clinical tissues. Following the manufacturer’s instructions, cDNA was synthesized using PrimeScript RT-polymerase (Vazyme). This was followed by the performance of RT-qPCR with SYBR-Green Premix (Qiagen GmbH) using Glyceraldehyde-3-phosphate dehydrogenase (GAPDH) as an internal control. The 2^−ΔΔCt^ method was performed to calculate the expressions of CASP8, miR-20a-5p, and SNHG14. The Kaplan–Meier method was performed to evaluate the prognostic value of CASP8 in BLCA.

### Statistical analyses

The mRNA levels of pyroptosis-related genes in BLCA versus normal tissues were analyzed using Student’s *t*-test. The Kaplan–Meier method was applied to draw the survival curve with the log-rank test calculating the *p* values, hazard ratio (HR), and 95% confidence interval (CI). The LASSO Cox regression analysis was performed to develop a pyroptosis-related prognostic signature. Pearson’s correlation test was performed to analyze the correlation between gene expression and immune cell infiltration and drug sensitivity.

## Results

### The expression and genetic variation landscape of pyroptosis-related genes in bladder cancer

**FIGURE 1:**
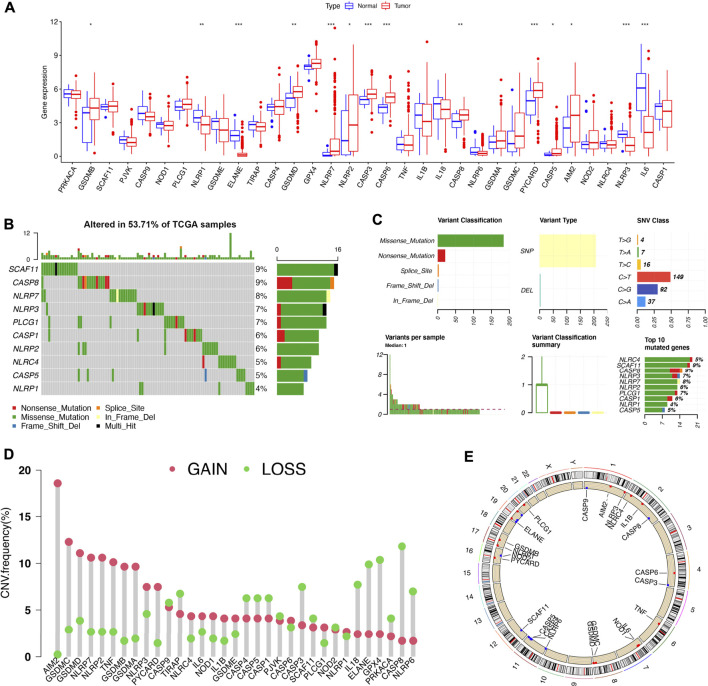
The expressions and genetic variations of PRGs in LUAD. **(A)** The expressions of PRGs in bladder cancer and bladder tissues. **(B,C)** The frequency and classification of PRGs’ genetic variations in bladder cancer. **(D,E)** The CNV variation frequency of PRGs and their corresponding location on 23 chromosomes in bladder cancer. The height of the column represents the alteration frequency. *p < 0.05; **p < 0.01; ***p < 0.001, PRG, pyroptosis-related gene; CNV, copy number variation.

Based on the TCGA bladder cancer cohort, the mRNA levels of 14 out of the 33 PRGs were either increased (GSDMB, GSDMD, NLRP7, NLRP2, CASP3, CASP6, CASP8, PYCARD, CASP5, and AIM2) or decreased (NLRP1, ELANE, NLRP3, and IL6) in bladder cancer versus bladder tissues (Figure 1A, all *p* < 0.05). This was followed by the analyses of copy number variations and somatic mutations of 33 PRGs in bladder cancer. We found that 53.71% of all bladder cancer samples showed genetic mutations in the TCGA bladder cancer cohort and SCAF11 was the most frequent mutation frequency gene, followed by CASP8 and NLRP7 (Figure 1B). The variant classification, variant type, and SNV class are shown in Figure 1C. In CNV alteration analysis, half of the 33 PRGs had copy number amplification while the other PRGs experienced CNV deletion (Figure 1D). Figure 1E shows the location of CNV alterations of most of the PRGs on human chromosomes.

### Gene Ontology and Kyoto Encyclopedia of Genes and Genomes pathway analyses

**FIGURE 2:**
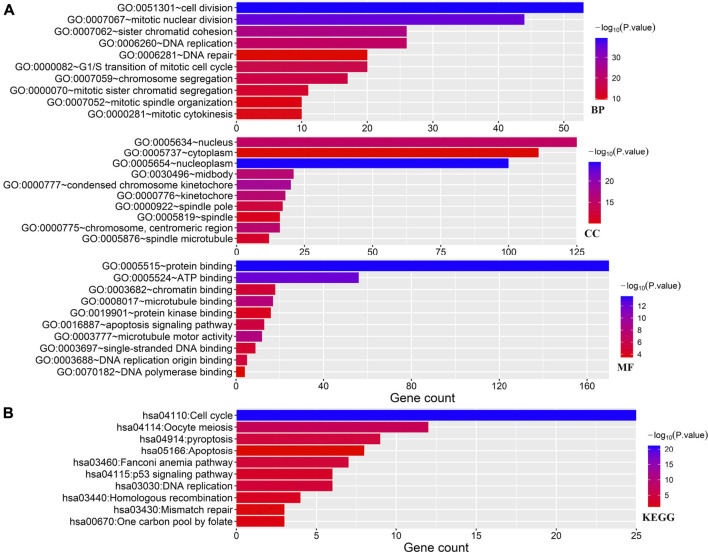
GO and KEGG analyses. **(A)** Enriched items in the GO analysis. **(B)** Enriched items in the KEGG analysis. GO, gene ontology; KEGG, Kyoto Encyclopedia of Genes and Genomes; BP, biological process; CC, cellular component; MF molecular function.

As shown in Figure 2A, the GO analysis demonstrated the enrichment of these PRGs in cell division, mitotic nuclear division, sister chromatid cohesion, G1/S transition of mitotic cell cycle, nucleus, cytoplasm, protein binding, ATP binding, apoptosis signaling pathway, and DNA replication origin-binding of these PRGs. Furthermore, the result of the Kyoto Encyclopedia of Genes and Genomes (KEGG) pathway analysis shown in Figure 2B, suggests that these PRGs were enriched in cell cycle, oocyte meiosis, pyroptosis, apoptosis, the p53 signaling pathway, and DNA replication.

#### Consensus clustering of pyroptosis-related genes in two clusters in bladder cancer

**FIGURE 3:**
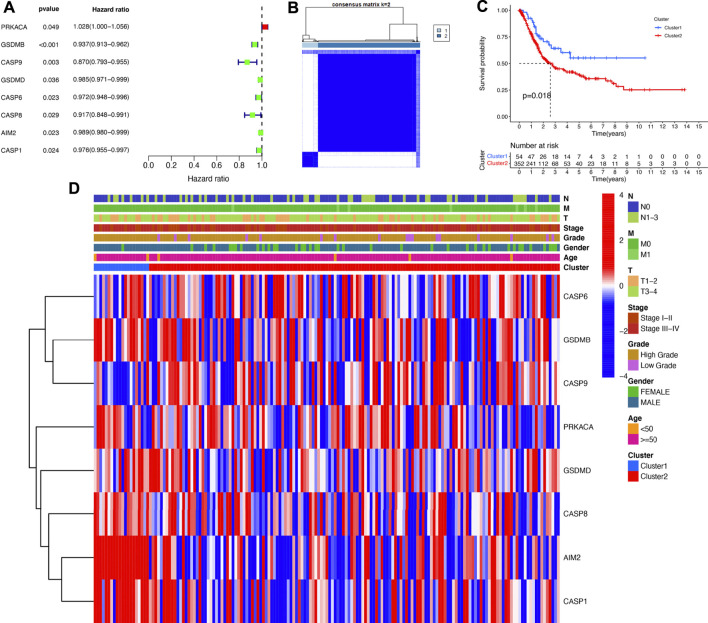
Consensus clustering of prognostic PRGs. **(A)** Univariate Cox regression analysis revealed that eight PRGs (PRKACA, GSDMB, CASP9, GSDMD, CASP6, CASP8, AIM2, and CASP1) were markedly correlated with the prognosis in bladder cancer. **(B)** Bladder cancer cases were divided into two clusters based on the consensus clustering matrix (k = 2). **(C)** Overall survival curve of bladder cancer patients in two clusters. **(D)** The difference of clinicopathological characteristics and gene expression in two clusters.

The Univariate Cox regression analysis revealed that eight PRGs (PRKACA, GSDMB, CASP9, GSDMD, CASP6, CASP8, AIM2, and CASP1) were significantly correlated with the prognosis in bladder cancer (Figure 3A). Based on these PRGs, a consensus clustering analysis was performed to cluster bladder cancer patients. As displayed in Figure 3B, the k = 2 was identified as the optimal clustering stability from k = 2 to 9 based on the similarity displayed by the PRG expressions and the TCGA bladder cancer cohort was separated into cluster 1 and cluster 2. The following survival analysis demonstrated a worse OS rated in cluster 2 than that of cluster 1 in bladder cancer (Figure 3C, *p* = 0.018). However, there was no difference in age, gender, tumor grade, clinical stage, and TMN stage between cluster 1 and cluster 2 (Figure 3D).

#### Consensus clustering correlated with different immune infiltration

**FIGURE 4:**
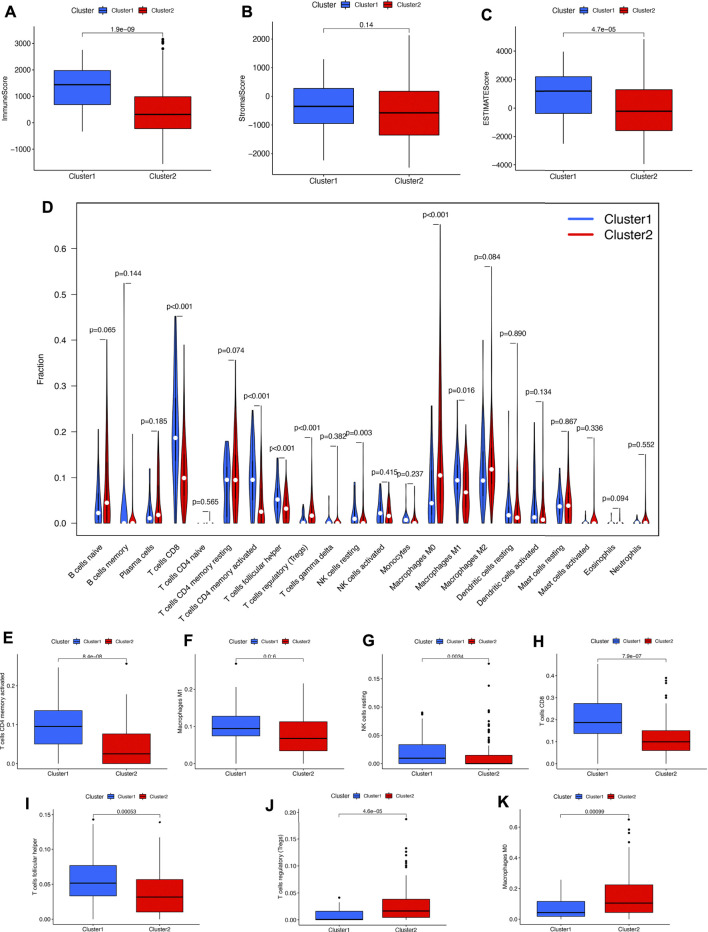
Cluster correlated with different immune infiltrations in bladder cancer. **(A–C)** Cluster1 correlated with high Immunoscore and ESTIMATEScore compared with those in cluster 2 in bladder cancer. **(D)** The difference of the abundance of 22 immune cells in the two clusters. **(E–K)** The infiltrating levels of CD4 memory-activated T cells, macrophage M1, NK resting cells, CD8 T cells, follicular helper T cells, Tregs, and macrophage M0 in two clusters.

To explore the potential functions of PRGs in the immune microenvironment of bladder cancer, we then analyzed the difference in immune infiltration. As shown in Figures 4A–C, the immuneScore (*p* = 1.9e^−9^) and ESTIMATEScore (*p* = 4.7e^−5^) in cluster 2 were lower than those in cluster 1 in bladder cancer. Figure 4D showed the infiltration landscape of 22 immune cells in cluster 1 and cluster 2 of bladder cancer. As expected, cluster 1 in bladder cancer was correlated with a higher abundance of CD4 memory-activated T cells, macrophage M1, NK resting cells, CD8 T cells, and follicular helper T cells and a higher abundance of T cells regulatory (Tregs) and macrophage M0 compared with cluster 2 (Figures 4E–K, all *p* < 0.05).

**FIGURE 5:**
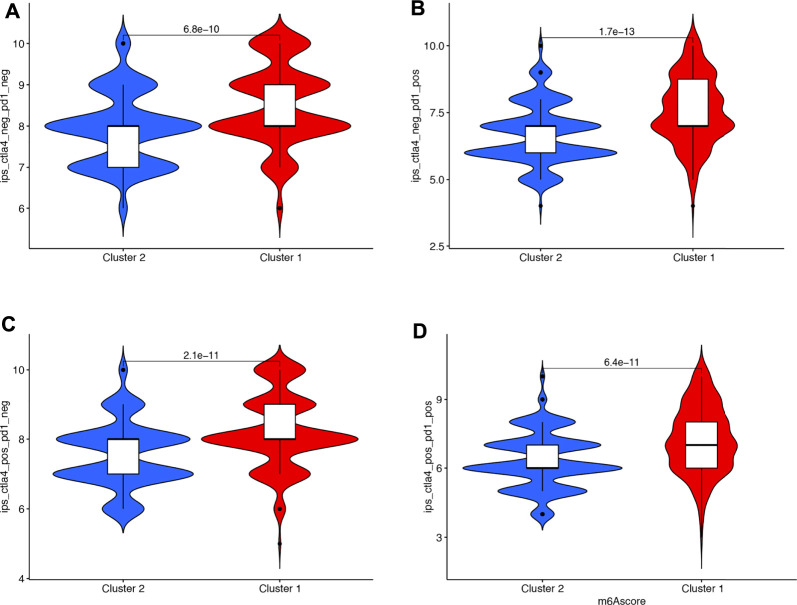
The IPS scores in cluster 1 and cluster 2 in bladder cancer. **(A–D)** A higher immunotherapy score in cluster 1 compared with cluster 2.

Immunotherapies represented by PD-1 and CTLA4 blockades have undoubtedly emerged as a major breakthrough in cancer therapy. We also compared the immunotherapy score between cluster 1 and cluster 2 in bladder cancer, which indicated a higher immunotherapy score in cluster 1 compared with cluster 2 (Figures 5A–D, all *p* < 0.05).

### Construction of a prognostic signature

**FIGURE 6:**
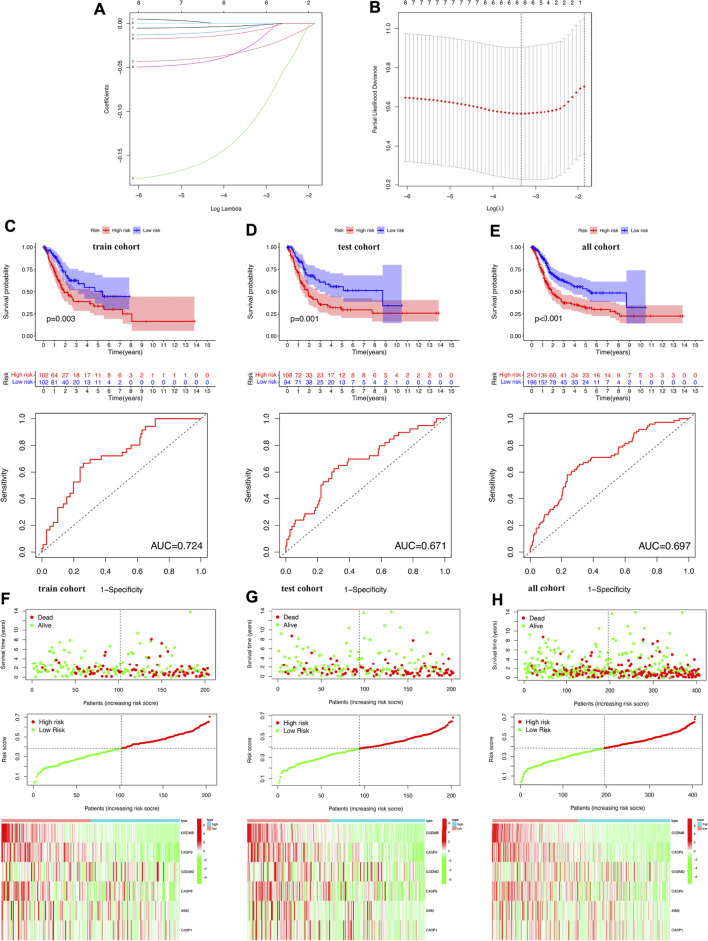
Construction of the prognostic signature in bladder cancer. **(A,B)** The coefficient and partial likelihood deviance of prognostic signature. **(C–E)** Overall survival curve in the high-/low-risk group and the ROC curve for prognosis predicting in the training cohort, test cohort, and bladder cancer cohort. **(F–H)** Riskscore distribution and survival status, the expression of prognostic PRGs in the training cohort, test cohort, and bladder cancer cohort.

**FIGURE 7:**
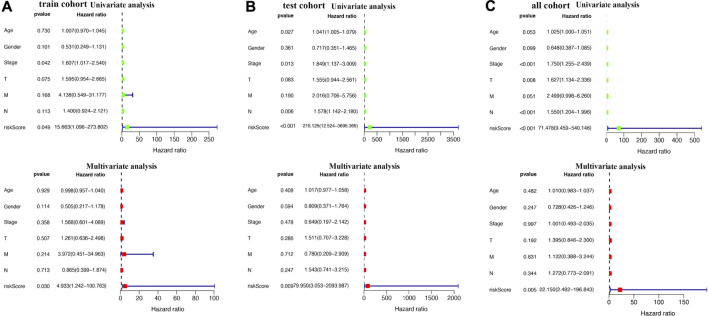
Univariate and multivariate Cox hazard ratio analysis considering riskScore and age, gender, clinical stage, pT stage, pN stage, and pM stage in the training cohort **(A)**, test cohort **(B)**, and all bladder cancer cohort **(C)**.

The LASSO Cox regression analysis was performed to construct a prognostic signature based on the prognostic PRGs identified previously. As a result, six PRGs were included in the LASSO Cox regression analysis and Figures 6A, B showed the coefficient and partial likelihood deviance of the prognostic signature. The TCGA bladder cancer cohort was divided into high- and low-risk subgroups with the median score as the cut-off value. Further prognosis analysis demonstrated a better prognosis in low-risk group patients than that in high-risk group patients in the training cohort, test cohort, and all TCGA cohorts, with an AUC of 0.724, 0.671, and 0.697, respectively (Figures 6C–E, all *p* < 0.05). These data revealed that these prognosis signatures had a favorable performance in predicting the prognosis of bladder cancer patients. Figures 6F–H show the riskScore distribution, patients’ survival status, and PRGs expression of training, test, and all TCGA cohorts, respectively. The Univariate and multivariable Cox regression analyses further demonstrated the risk score as an independent prognostic factor for bladder cancer patients in training, test, and all TCGA cohorts (Figures 7A–C, all *p* < 0.05).

#### RiskScore correlated with different clinical characters and immune cell infiltration in bladder cancer

**FIGURE 8:**
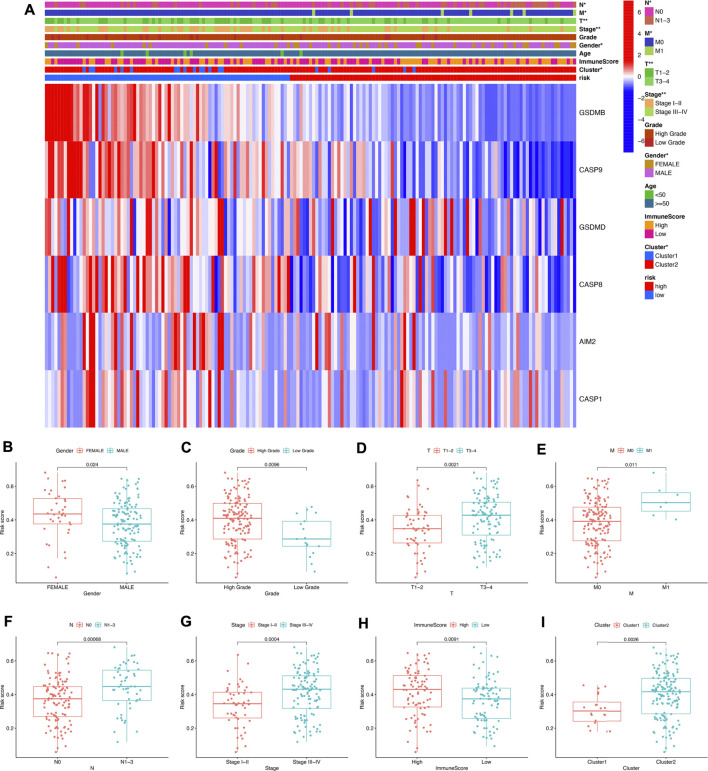
Risk score correlated with clinicopathological features in bladder cancer. **(A)** The heatmap revealed the difference of clinical characters between the high- and low-risk groups in bladder cancer. The risk score in different gender **(B)**, tumor grade **(C)**, pT stage **(D)**, pM stage **(E)**, pN stage **(F)**, clinical stage **(G)**, immunoScore **(H),** and cluster **(I)** of bladder cancer patients. *p < 0.05, **p < 0.01.

**FIGURE 9:**
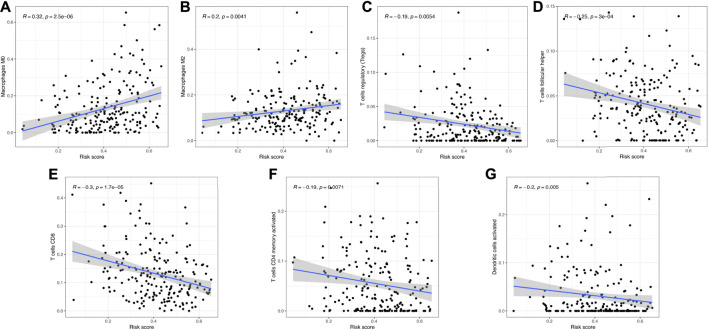
Correlation between the risk score and immune cell infiltration. **(A,B)** Risk score showed positive correlation with the abundance of the macrophages M0 and M2. **(C–G)** As the risk score increased, the abundance of regulatory T cells, follicular helper T cells, CD8 T cells, CD4 memory activated T cells, and activated dendritic cells decreased.

The heatmap in Figure 8A reveals the difference of clinical characters between the high- and low-risk groups in bladder cancer. To be more specific, female (Figure 8B) and high-tumor grade (Figure 8C) patients had a high-risk score versus male and low-tumor grade patients (*p* < 0.05). Moreover, low risk score was correlated with those patients in pT1-T2 stage, pM0 stage, pN0 stage, and early clinical stage in bladder cancer (Figures 8D–G, all *p* < 0.05). We also found that bladder cancer patients with a high immune score (Figure 8H) or in the cluster 2 (Figure 8I) subgroup had a high-risk score (*p* < 0.05). Figure 9 reveals the correlation between risk score and immune cell infiltration. These data indicate a positive correlation between risk score and the infiltration levels of the macrophage M0 (cor = 0.32) and macrophage M2 (cor = 0.2) (Figures 9A,B, *p* < 0.05). As the risk score increases, the abundance of regulatory T cells, follicular helper T cells, CD8 T cells, CD4 memory-activated T cells, and activated dendritic cells decreases (Figures 9C–G, *p* < 0.05).

### Pyroptosis-related genes correlated with immune cell infiltration in bladder cancer

We also analyzed the correlation between the aforementioned six prognostic signature genes and immune cell infiltration in bladder cancer. The result demonstrated a positive correlation between the expressions of CASP1 and GSDMD and the abundance of B cells, CD4^+^ T cells, CD8^+^ T cells, neutrophils, macrophages, and dendritic cells (Supplementary Figures S1A,B, all *p* < 0.05). As the expressions of CASP8 and AIM2 increased, the abundance of B cells, CD8^+^ T cells, neutrophils, and dendritic cells increased (Supplementary Figures S1C,D, all *p* < 0.05). We also found a negative correlation between the expressions of CASP9 and GSDMB and the infiltration level of most of immune cells (Supplementary Figures S1E,F). Interestingly, the somatic copy number alteration of the six prognostic signature genes could inhibit immune cell infiltration to some extent (Supplementary Figure S2).

### Construction of a regulatory axis of mRNA–miRNA–lncRNA

**FIGURE 10:**
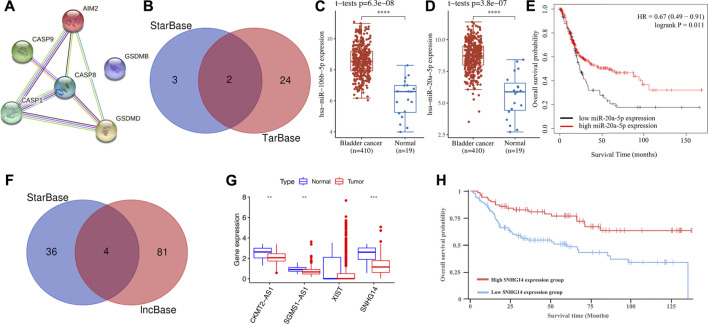
Construction of the lncRNA–miRNA–mRNA regulatory axis. **(A)** A PPI network identified CASP8 as the hub gene. **(B)** miRNA targets predicted by StarBase and TarBase. **(C,D)** The expression of miR-106b-5p and miR-20a-5p in bladder cancer and normal tissues. **(E)** The prognostic value of miR-20a-5p in bladder cancer. **(F)** lncRNA targets predicted by lncBase and StarBase. **(G)** The expressions of lncRNA CKMT2-AS1, SGMS1-AS1, XIST, and SNHG14 in bladder cancer tissues and normal tissues. **(H)** Bladder cancer patients with low SNHG14 expression had a poor overall survival.

We then constructed a PPI network and CASP8 was identified as the hub gene for further analysis (Figure 10A). To clarify the potential molecular mechanism of CASP8 in bladder cancer, we then constructed a regulatory axis of mRNA–miRNA–lncRNA. Based on the results of TarBase and StarBase, miR-106b-5p and miR-20a-5p were identified as potential miRNA targets for CASP8 (Figure 10B). Interestingly, the expressions of miR-106b-5p (*p* = 6.3e^−8^) and miR-20a-5p (*p* = 3.8e^−7^) in bladder cancer were elevated in bladder cancer tissues (Figures 10C,D). However, the prognosis analysis only suggested miR-20a-5p as a prognosis biomarker and correlated with a better overall survival (Figure 10E). We then detected the upstream lncRNA targets of miR-20a-5p. Four lncRNAs, including CKMT2-AS1, SGMS1-AS1, XIST, and SNHG14 were suggested as the upstream targets (Figure 10F). Moreover, further expression analyses suggested a downregulation of CKMT2-AS1, SGMS1-AS1, and SNHG14 (Figure 10G, all *p* < 0.05) in bladder cancer. However, only lncRNA SNHG14 was suggested as a prognostic biomarker and was correlated with a better overall survival in bladder cancer (Figure 10H, *p* = 0.0361). Therefore, the lncRNA SNHG14/miR-20a-5p/CASP8 regulatory axis may play a vital role in the progression of bladder cancer. However, further study should be performed to provide clear experimental evidence and verify this result.

#### Validation of the expression of SNHG14/miR-20a-5p/CASP8 and the overall survival of CASP8 in bladder cancer

**FIGURE 11:**
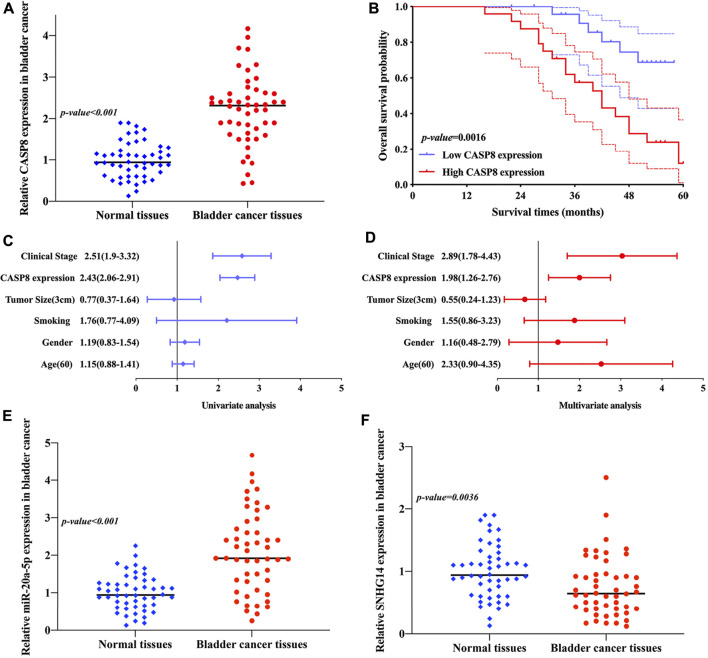
Validation of the expression of SNHG14/miR-20a-5p/CASP8 and the overall survival of CASP8 in bladder cancer. **(A)** CASP8 expression was upregulated in bladder cancer versus bladder tissues. **(B)** Bladder cancer patients with high CASP8 expression had a poor overall survival. **(C,D)** Univariate and multivariate analyses demonstrated the CASP8 expression and clinical stage as prognostic factors affecting the overall survival of bladder cancer patients. **(E,F)** Upregulation of miR-20a-5p and downregulation of lncRNA SNHG14 were obtained in bladder cancer versus bladder tissues.

We then explored the expression of CASP8 in bladder cancer. As expected, the result of qRT-PCR revealed that CASP8 expression was upregulated in bladder cancer tissue versus bladder tissue (*p* < 0.001, Figure 11A). Moreover, the Kaplan–Meier curve revealed that bladder cancer patients with high CASP8 expression had a poor overall survival (*p* = 0.0016, Figure 11B). Furthermore, the univariate and multivariate analyses suggested that CASP8 expression and clinical stage as independent factors affected the overall survival of bladder cancer patients (Figures 11C,D). Moreover, these data also demonstrated the upregulation of miR-20a-5p (*p* < 0.001, Figure 11E) and downregulation of lncRNA SNHG14 (*p* = 0.0036, Figure 11F) in bladder cancer tissue versus bladder tissue.

## Discussion

Pyrolysis is a pro-inflammatory form of cell death, involved in the pathogenesis of many diseases, including cardiovascular diseases, diabetic nephropathy, and malignancies (Liu et al., 2017; Fang et al., 2020). The mechanisms of pyrolysis in cancer development and therapy are complicated. A large number of inflammatory cytokines released by pyroptosis could stimulate the transformation of normal cells into tumor cells. However, pyroptosis could promote the death of tumor cells, making pyroptosis a potential therapeutic target for cancer (Karki and Kanneganti, 2019; Ye et al., 2021). Recent studies suggest the involvement of pyroptosis in the proliferation, invasion, and metastasis of cancer (Xia et al., 2019). Some scholars even demonstrated pyroptosis as a novel strategy for cancer treatment due to its effects to all the stages of carcinogenesis (Ruan et al., 2020). Moreover, accumulating evidence suggested PRGs as prognosis biomarkers for some types of cancers, including ovarian cancer and lung adenocarcinoma (Wei et al., 2020; Ye et al., 2021). The pyroptosis-related gene CASP8 is a molecular switch controlling tumor cell pyroptosis and it could promote the progression of glioma (Fritsch et al., 2019; Chen et al., 2021a). Pyroptosis-related gene GSDMB is a prognostic biomarker in RCC and is involved in many biological processes and immune infiltration (Cui et al., 2021; Pan et al., 2022). However, the prognostic value of PRGs and their association with immune infiltration in bladder cancer have not yet been fully elucidated.

Here, two distinct clusters of bladder cancer were identified based on eight PRGs and these two clusters had conspicuously different tumor microenvironment (TME) cell infiltration characterizations. Cluster 1 of the TCGA bladder cancer cohort was characterized by the abundant infiltration of immune cells, corresponding to the immune-inflamed phenotype while cluster 2 was characterized by the suppression of immunity, corresponding to the immune-desert phenotype. The immune-inflamed phenotype was referred to as “hot tumor” and associated with plentiful immune cell infiltrations in TME (Zhang et al., 2020). The immune-desert phenotype, so-called “cold tumor”, was typical of immune tolerance and ignorance, and activated T-cell deficiency (Kim and Chen, 2016; Bonaventura et al., 2019). Cold tumor is a big treatment challenge for cancer immunotherapy (Bonaventura et al., 2019). In our study, the result also suggested a low immunotherapy score in cluster 2 compared with cluster 1. It was not surprising that cluster 2 of the TCGA bladder cancer cohort had poorer prognosis. Actually, the consensus clustering analysis and molecular subtype classification of cancer with distinct biological characteristics were crucial for the precise treatment of malignancies. The molecular classification of breast cancer played a vital role in the development of precise treatment and clinical practice (Zhao et al., 2020). Another SUMOylation pattern could predict the prognosis and tumor microenvironment infiltration characterization in bladder cancer (Xia et al., 2022b). Moreover, Qi-Dong et al. developed a ferroptosis pattern in bladder cancer that could guide the clinical therapeutic strategy (Xia et al., 2022c).

Another vital finding of our study was that we constructed a prognostic signature using the LASSO Cox regression analysis with six PRGs with prognostic value (GSDMB, CASP9, GSDMD, CASP8, AIM2, and CASP1) and this prognostic signature showed a favorable performance for predicting the prognosis of bladder cancer patients. In fact, several prognostic signatures had been developed for bladder cancer. Using seven immune-related genes, Qiu et al. (2020) constructed an individualized prognostic signature for bladder cancer. Another 18 DNA methylation-related gene signatures showed a favorable predictive performance in the prognostic prediction for bladder cancer (Liu et al., 2021). To predict the OS and therapeutic responses in bladder cancer, Wu et al. (2020) constructed a TP53-associated immune prognostic signature. Moreover, another study developed a prognostic signature for bladder cancer based on twelve immune infiltration-related lncRNAs (Liu et al., 2022). In our study, we firstly identified a pyroptosis-related prognostic signature for bladder cancer and we could predict the prognosis of each patient by calculating the risk score. Another finding of our study was that the risk score and pyroptosis-related prognostic signature genes were correlated with different clinical characters and immune infiltration in bladder cancer. A previous study revealed that pyroptosis influences the tumor immune microenvironment in bladder cancer (Chen et al., 2021b). The level of immune infiltration was correlated with the prognosis and immunotherapy benefit of bladder cancer (Wang et al., 2020). In the current study, riskScore and pyroptosis-related prognostic signature were correlated with immune infiltration in bladder cancer. Whether they could predict the immunotherapy response of bladder cancer needs to be further studied.

By constructing an mRNA–miRNA–lncRNA network, we identified an lncRNA SNHG14/miR-20a-5p/CASP8 regulatory axis, which may play a vital role in the development of bladder cancer. Interestingly, previous studies had also highlighted the vital role of lncRNA NHG14 and miR-20a-5p in bladder cancer. Upregulation of SNHG14 promoted the proliferation and cell cycle progression in bladder cancer by regulating miRNA-150-5p (Li et al., 2019). Moreover, miR-20a-5p acted as a prognosis marker in bladder cancer and it could accelerate tumor cell proliferation and migration (Yin et al., 2019; Zhong et al., 2020). The result of our study also suggested that miR-20a-5p was a prognosis marker of bladder cancer patients. All these pieces of evidence revealed that the lncRNA SNHG14/miR-20a-5p/CASP8 regulatory axis may also play vital functions in the progression of bladder cancer. Further *vivo* and *vitro* experiments are necessary to verify these results.

Some limitations could be found in our study. It would be better to verify all the results using the GEO cohort. The expression and prognosis value of PRGs should be verified using clinical tissues. Further *vivo* and *vitro* experiments are necessary to verify the lncRNA SNHG14/miR-20a-5p/CASP8 regulatory axis in bladder cancer.

## Conclusion

By comprehensive analysis of the prognostic value and immune infiltrates of PRGs in bladder cancer, we identified a pyroptosis-related prognostic signature and the lncRNA SNHG14/miR-20a-5p/CASP8 regulatory axis for bladder cancer. Further study should be conducted to verify this result.

## Data Availability

The datasets presented in this study can be found in online repositories. The names of the repository/repositories and accession number(s) can be found in the article/Supplementary Material.
