# Supplementary material for: Identification and verification of the pyroptosis-related prognostic signature and its associated regulatory axis in bladder cancer
Source: Front Cell Dev Biol. 2022 Aug 31;10:912008. doi: 10.3389/fcell.2022.912008 (PMC9470881; doi:10.3389/fcell.2022.912008)
Supplement: Supplementary file 2 [file Table1.DOCX]

Supplementary Table 1. The clinicopathologic features of bladder cancer patients in TCGA cohort.

| Clinical characters | Number |
| --- | --- |
| Gender  Male  Female | 304  108 |
| Age (years, x ± s) | 68.1 ± 10.5 |
| Grade  High  Low  Unknown | 388  21  3 |
| TNM stage  I  II  III  IV  Unknown | 2  131  141  136  2 |
| T  T0  T1  T2  T3  T4  Unknown | 1  3  120  196  59  33 |
| N  N0  N1  N2  N3  Unknown | 237  47  76  8  41 |
| M  M0  M1  Unknown | 196  11  205 |

Supplementary Table 2. 33 pyroptosis-related genes

| Genes | Full-names |
| --- | --- |
| AIM2 | Absent in melanoma 2 |
| CASP1 | cysteine-aspartic acid protease-1 |
| CASP3 | cysteine-aspartic acid protease-3 |
| CASP4 | cysteine-aspartic acid protease-4 |
| CASP5 | cysteine-aspartic acid protease-5 |
| CASP6 | cysteine-aspartic acid protease-6 |
| CASP8 | cysteine-aspartic acid protease-8 |
| CASP9 | cysteine-aspartic acid protease-9 |
| ELANE | elastase, neutrophil expressed |
| GPX4 | glutathione peroxidase 4 |
| GSDMA | gasdermin A |
| GSDMB | gasdermin B |
| GSDMC | gasdermin C |
| GSDMD | gasdermin D |
| GSDME | gasdermin E |
| IL18 | interleukin 18 |
| IL1B | interleukin 1 beta |
| IL6 | interleukin 6 |
| NLRC4 | NLR family CARD domain containing 4 |
| NLRP1 | NLR family pyrin domain containing 1 |
| NLRP2 | NLR family pyrin domain containing 2 |
| NLRP3 | NLR family pyrin domain containing 3 |
| NLRP6 | NLR family pyrin domain containing 6 |
| NLRP7 | NLR family pyrin domain containing 7 |
| NOD1 | nucleotide binding oligomerization domain containing 1 |
| NOD2 | nucleotide binding oligomerization domain containing 2 |
| PJVK | pejvakin/deafness, autosomal recessive 59 |
| PLCG1 | phospholipase C gamma 1 |
| PRKACA | protein kinase cAMP-activated catalytic subunit alpha |
| PYCARD | PYD and CARD domain containing |
| SCAF11 | SR-related CTD associated factor 11 |
| TIRAP | TIR domain containing adaptor protein |
| TNF | tumor necrosis factor |
