# Supplementary figures and images for: Identification and verification of the pyroptosis-related prognostic signature and its associated regulatory axis in bladder cancer
Source: Front Cell Dev Biol. 2022 Aug 31;10:912008. doi: 10.3389/fcell.2022.912008 (PMC9470881; doi:10.3389/fcell.2022.912008)

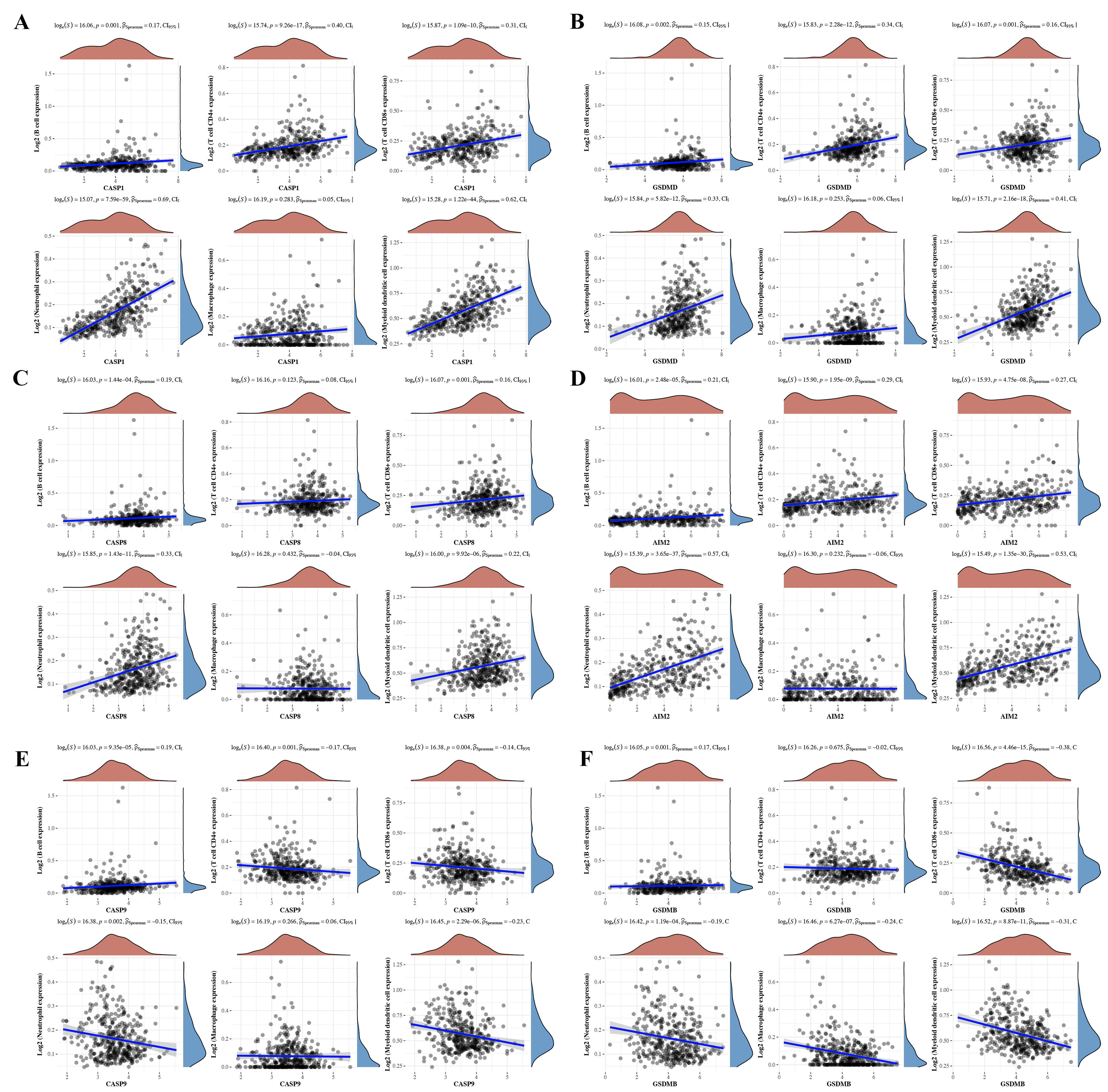

Supplement: Supplementary file 3 [file Image1.JPEG]

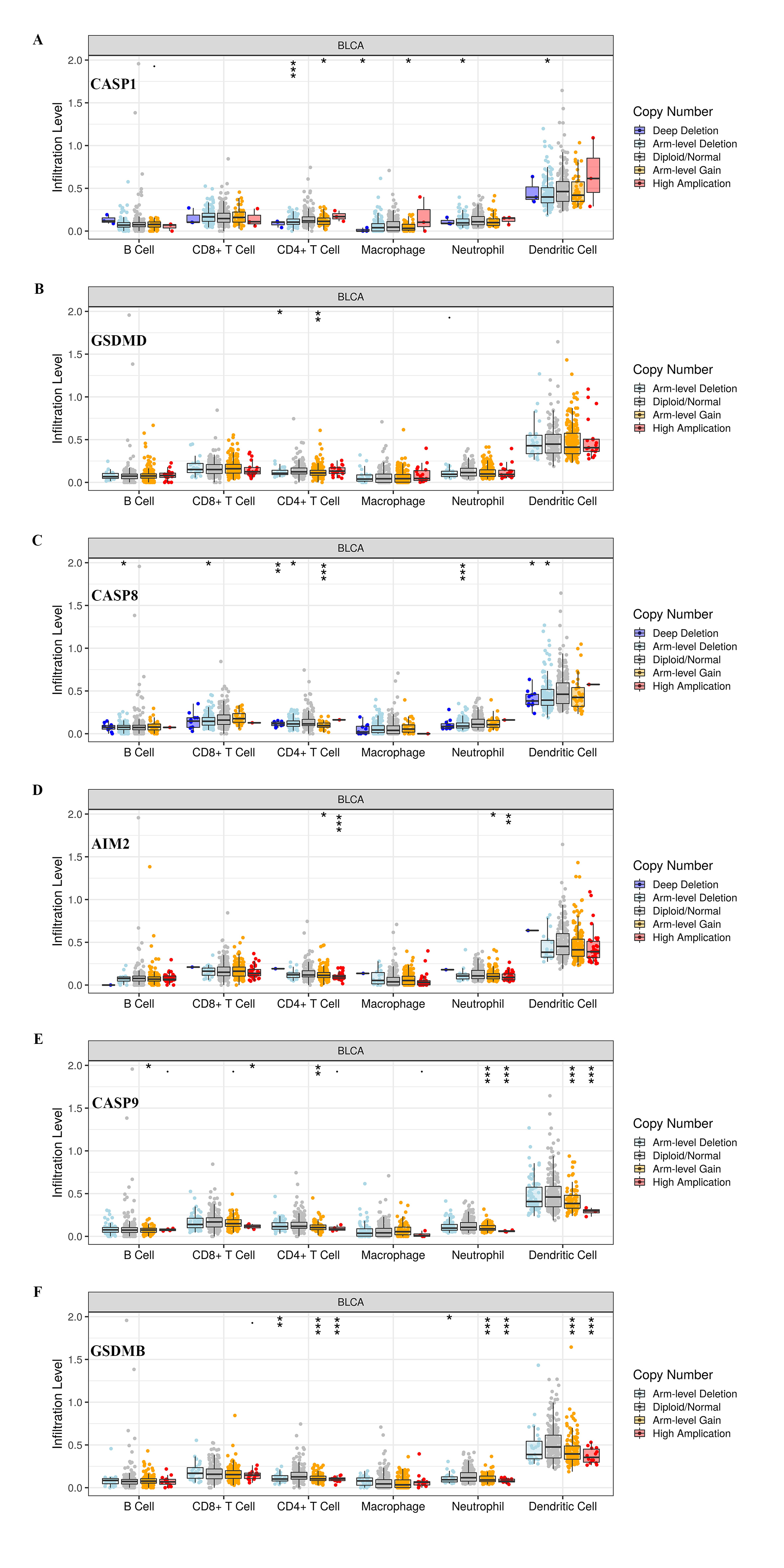

Supplement: Supplementary file 4 [file Image2.JPEG]
